# Long noncoding RNA SNHG12 promotes tumour progression and sunitinib resistance by upregulating CDCA3 in renal cell carcinoma

**DOI:** 10.1038/s41419-020-2713-8

**Published:** 2020-07-08

**Authors:** Yuenan Liu, Gong Cheng, Ziwei Huang, Lin Bao, Jingchong Liu, Cheng Wang, Zhiyong Xiong, Lijie Zhou, Tianbo Xu, Di Liu, Hongmei Yang, Ke Chen, Xiaoping Zhang

**Affiliations:** 1 Department of Urology, Union Hospital, Tongji Medical College, Huazhong University of Science and Technology, No. 1277 Jiefang Avenue, 430022 Wuhan, China; 2 Department of Breast and Thyroid Surgery, Union Hospital, Tongji Medical College, Huazhong University of Science and Technology, No. 1277 Jiefang Avenue, 430022 Wuhan, China; 3 Department of Pathogenic Biology, School of Basic Medicine, Huazhong University of Science and Technology, No. 13 Hangkong Road, 430030 Wuhan, China

**Keywords:** Renal cell carcinoma, Mechanisms of disease, Long non-coding RNAs

## Abstract

Renal cell carcinoma (RCC) is one of the most frequently observed malignant tumours in the urinary system and targeted drug resistance is quite common in RCC. Long noncoding RNA SNHG12 (lncRNA SNHG12) has emerged as a key molecule in numerous human cancers, but its functions in renal cell carcinoma (RCC) sunitinib resistance remain unclear. In this study, we found SNHG12 was highly expressed in RCC tissues and in sunitinib-resistant RCC cells and was associated with a poor clinical prognosis. SNHG12 promoted RCC proliferation, migration, invasion and sunitinib resistance via CDCA3 in vitro. Mechanically, SNHG12 bound to SP1 and prevented the ubiquitylation-dependent proteolysis of SP1. Stabilised SP1 bound to a specific region in the promoter of CDCA3 and increased CDCA3 expression. Furthermore, in vivo experiments showed that SNHG12 increased tumour growth and that knocking down SNHG12 could reverse RCC sunitinib resistance. Our study revealed that the lncRNA SNHG12/SP1/CDCA3 axis promoted RCC progression and sunitinib resistance, which could provide a new therapeutic target for sunitinib-resistant RCC.

## Introduction

Renal cell carcinoma (RCC) is one of the most frequently observed malignant tumours in the urinary system^1^. Among the various pathological types of RCC, ~70–80% are clear cell renal cell carcinoma (ccRCC)^2^. It is estimated that the number of new cases of RCC will be 73,820, and a total of 14,770 mortalities will be reached in the USA in 2019^3^. The diagnosis of RCC at an early stage is not easy because the initial symptoms are usually not obvious. In fact, for ~30% of RCC patients, cancer has already metastasised at first diagnosis^4^. Although targeted drugs, such as sunitinib, have been used to treat advanced RCC patients, unfortunately, drug resistance develops within 6–15 months, and the overall survival remains unsatisfactory^5–7^. As a result, it is necessary to elucidate the potential mechanisms underlying RCC progression and drug resistance.

Long noncoding RNAs (lncRNAs) are defined as a group of RNAs with a minimum length of 200 nucleotides that do not encode proteins^8^. However, studies have demonstrated their significant roles in the regulation of transcription and translation of protein-coding genes^9–11^. Based on these functions, lncRNAs are involved in multiple oncogenic processes, including tumour cell proliferation, metastasis, apoptosis inhibition and drug resistance^12–14^. Small nucleolar RNA host gene 12 (SNHG12) has been increasingly reported to participate in diverse cancers^15^. It is believed that SNHG12 could act as a molecular sponge of microRNAs to promote tumour proliferation, metastasis and epithelial-mesenchymal transition^16,17^. Furthermore, SNHG12 is also involved in multidrug resistance^18,19^. However, the functions and mechanisms of SNHG12 in RCC remain unclear.

In the present study, we screened lncRNAs involved in sunitinib-resistant RCC by bioinformatics analysis. We found that lncRNA SNHG12 was highly expressed and could be a diagnostic and prognostic biomarker of RCC. In addition, overexpression of SNHG12 promoted RCC progression and sunitinib resistance both in vitro and in vivo. By further exploration, we determined that SNHG12 could upregulate CDCA3 by stabilising the transcription factor SP1. Therefore, our study describes a novel SNHG12/SP1/CDCA3 axis that might be a promising therapeutic target for RCC.

## Results

### SNHG12 was overexpressed and indicated a poor clinical prognosis in RCC

**Fig. 1:**
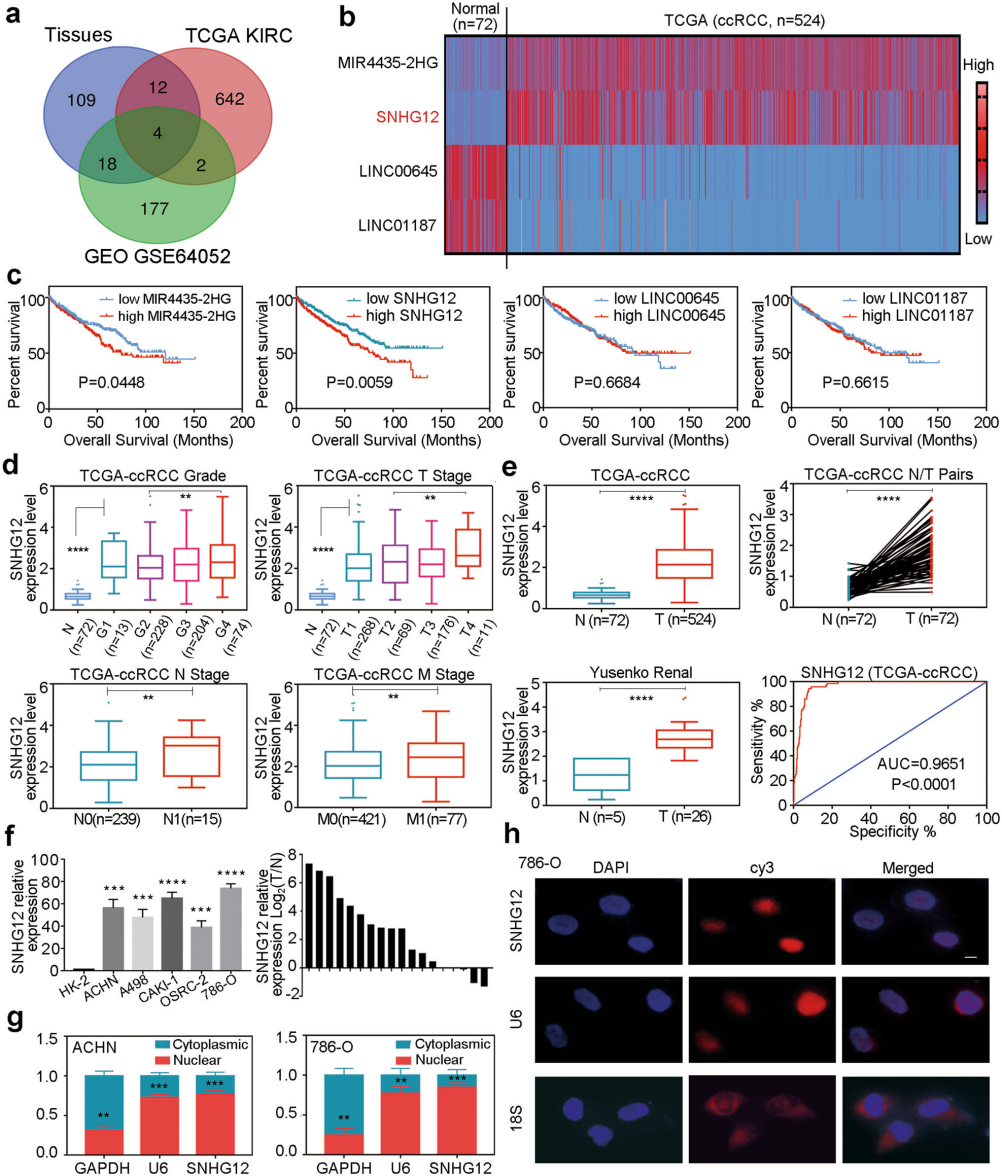
SNHG12 was overexpressed and indicated a poor clinical prognosis in RCC. **a** A Venn diagram of three independent lncRNA differential analysis. Blue: RNA sequence data of 3 RCC tissues and paired normal tissues. Red: TCGA-KIRC database. Green: GEO database (GSE64052). **b** The expression levels of MIR4435-2HG, SNHG12, LINC00645 and LINC01187 in 72 normal tissues and 524 ccRCC tissues in ccRCC based on data from the TCGA database. **c** The Kaplan–Meier curves of MIR4435-2HG, SNHG12, LINC00645 and LINC01187 in ccRCC for overall survival (OS). **d** The high expression of SNHG12 was related with various clinicopathological factors: G grade, T stage, lymph node metastasis and distant metastases. **e** The expression level of SNHG12 was higher in ccRCC tissues than in normal tissues in TCGA database and in Yusenko renal database. The ROC (receiver operating characteristic) curves of SNHG12 (AUC = 0.9651; *p* < 0.0001). **f** The expression levels of SNHG12 in five RCC cell lines (786-O, A498, ACHN, CAKI-1 and OSRC-2) and normal cell line (293T). The expression levels of SNHG12 in 20 RCC tissues and paired normal tissues. **g** Localisation of SNHG12 was assessed by PCR in ACHN and 786-O cells. U6 and GAPDH were used as positive controls for nuclear RNA and cytoplasmic RNA, respectively. **h** The distribution of SNHG12 was analysed by FISH in 786-O cells. 18S and U6 showed cytoplasm and nucleus, respectively. Scale bars, 10 μm. *P < 0.05, **P < 0.01, ***P < 0.001, ****P < 0.0001. Error bars indicate mean ± SD.

Accumulating studies have demonstrated that lncRNAs play important roles in tumour progression and drug resistance. We sequenced 3 pairs of RCC and adjacent normal tissues and analysed the differential expression of lncRNAs. We also studied the differential expression of lncRNAs between tumour and normal tissues from the TCGA-KIRC database. Moreover, differentially expressed lncRNAs between sunitinib-resistant RCC and sunitinib-sensitive RCC were determined from the GEO database (GSE64052^20^). From the intersection of these three datasets, 4 lncRNAs were obtained, two of which were upregulated and two were downregulated (Fig. 1a, b). For further analysis, we mined data in the TCGA-KIRC database and in Yusenko Renal^21^ from the Oncomine database. Among the four identified lncRNAs, only the SNHG12 expression level was correlated with the overall survival time, tumour grade and tumour stage of ccRCC patients (Fig. 1c, d and Supplementary Fig. 1). Higher SNHG12 expression indicated shorter survival time and higher tumour grade and stage (Table 1). Univariate and multivariate analyses were performed demonstrating that SNHG12 was one of the independent prognostic markers of ccRCC (Table 2). SNHG12 was expressed at a higher level in tumour tissues than in normal tissues, and the receiver operating characteristic (ROC) curve showed that SNHG12 could be used as a diagnostic biomarker for ccRCC (Fig. 1e).

**Table 1:** Correlation between SNHG12 mRNA expression and clinicopathological parameters of ccRCC patients.

Parameter		Number	SNHG12 mRNA expression		*P* value
			Low (*n* = 250)	High (*n* = 250)	
Age (years)	<60	233	111	122	0.370
	≥60	267	139	128	
Gender	Female	173	108	65	<0.001
	Male	327	142	185	
T stage	T1 or T2	339	184	155	0.007
	T3 or T4	161	66	95	
N stage	N0 or NX	488	248	240	0.036
	N1	12	2	10	
M stage	M0 or MX	447	239	208	<0.001
	M1	53	11	42	
G grade	G1 or G2orGx	246	137	109	0.016
	G3 or G4	254	113	141	
TNM stage	I + II	321	176	145	0.005
	III + IV	179	74	105	

*TNM* tumour-node-metastasis, *SNHG12* small nucleolar RNA host gene 12, *ccRCC* clear cell renal cell carcinoma.

**Table 2:** Univariate and multivariate analyses of SNHG12 mRNA level and patient survival.

Variables	Univariate analysis			Multivariate analysis^c^		
	HR^a^	95% CI^b^	*P* value	HR	95% CI	*P* value
Overall survival (*n* = 500)						
SNHG12 low (*n* = 250) high (*n* = 250)	2.021	1.449–2.819	0.000	1.510	1.060–2.152	0.022
Age (years) <60 (*n* = 233) ≥60 (*n* = 267)	2.011	1.429–2.829	0.000	1.807	1.277–2.555	0.001
Gender female (*n* = 173) male (*n* = 327)	0.962	0.689–1.343	0.821	–	–	–
T stage T1 or T2 (*n* = 339) T3 or T4 (*n* = 161)	2.963	2.147–4.088	0.000	1.660	1.156–2.384	0.006
N stage N0 or NX (*n* = 488) N1 (*n* = 12)	3.184	1.558–6.504	0.001	1.520	0.724–3.192	0.268
M stage M0 or MX (*n* = 447) M1 (*n* = 53)	4.855	3.410–6.912	0.000	2.961	1.990–4.404	0.000
G grade G1 or G2 or GX (*n* = 246) G3 or G4 (*n* = 254)	2.562	1.797–3.651	0.000	1.806	1.246–2.620	0.002

^a^Hazard ratio, estimated from Cox proportional hazard regression model.

^b^Confidence interval of the estimated HR.

^c^Multivariate models were adjusted for T, N, M classification, age and gender.

To verify the above results, RCC cell lines and more tumour tissues were added to the study. As shown in Fig. 1f, SNHG12 expression levels were significantly higher in RCC cells and tissues. According to the cellular fractionation assay of RNA, SNHG12 was mainly located in the nucleus in RCC cells (Fig. 1g). Furthermore, RNA fluorescence in situ hybridisation assays also produced the same results (Fig. 1h and Supplementary Fig. 2). Therefore, we found that nucleus-located lncRNA SNHG12 was highly expressed in RCC and was a predictor of a poor clinical prognosis.

### SNHG12 increased the proliferation, invasion and migration of RCC cells in vitro

**Fig. 2:**
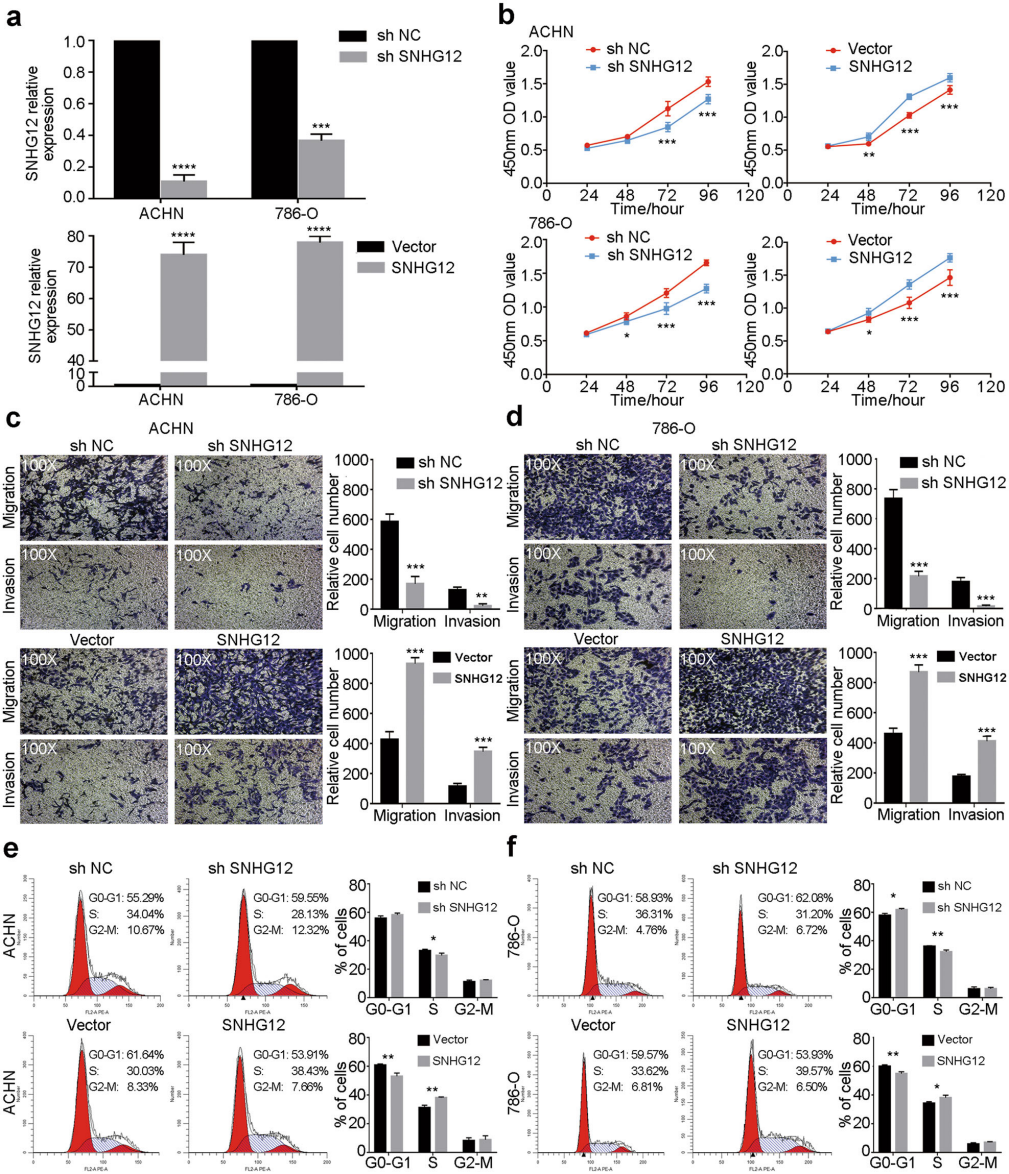
SNHG12 increased the proliferation, invasion and migration of RCC cells in vitro. **a** qRT-PCR assays were applied to analyse the expression level of SNHG12 after transfection by sh SNHG12 or SNHG12 overexpression vector for 24 h in ACHN or 786-O cells. **b** Cell viability of ACHN and 786-O cells after knocking down or overexpressing SNHG12 was determined using CCK8 assays. **c**, **d** Transwell assays were performed in transfected ACHN and 786-O cells to evaluate cell migration and invasion ability (Magnification: ×100). **e**, **f** Cell cycle distribution was analysed by PI staining in ACHN and 786-O cells after transfection by sh RNA or overexpression plasmid for 48 h. *P < 0.05, **P < 0.01, ***P < 0.001, ****P < 0.0001. Error bars indicate mean ± SD.

To determine the biological function of SNHG12 in RCC cells, we transfected short hairpin RNA (shRNA) or the pcDNA3.1 vector to knockdown or overexpress SNHG12 in 786-O and ACHN cells. Quantitative reverse transcription PCR (qRT-PCR) was used to determine the effect of transfection (Fig. 2a). CCK8 assays showed that knocking down SNHG12 expression significantly reduced the proliferation ability of 786-O cells and ACHN cells; conversely, overexpressing SNHG12 increased cell proliferation (Fig. 2b). Transwell assays were used to assess the migration and invasion ability of cells. SNHG12 knockdown resulted in a lower 786-O cell and ACHN cell migration and invasion rate, while SNHG12 overexpression showed the opposite results (Fig. 2c, d). In addition, cell cycle assays suggested that knockdown of SNHG12 inhibited cell cycle progression from G0/G1 phase to S phase. More cells were arrested at G0/G1 phase, so cell proliferation decreased. In contrast, overexpression of SNHG12 promoted entry of more cells into S phase so that the cell proliferation rate increased (Fig. 2e, f). In summary, SNHG12 increased the proliferation, invasion and migration of RCC cells.

### SNHG12 upregulated the expression of CDCA3 in RCC cells

**Fig. 3:**
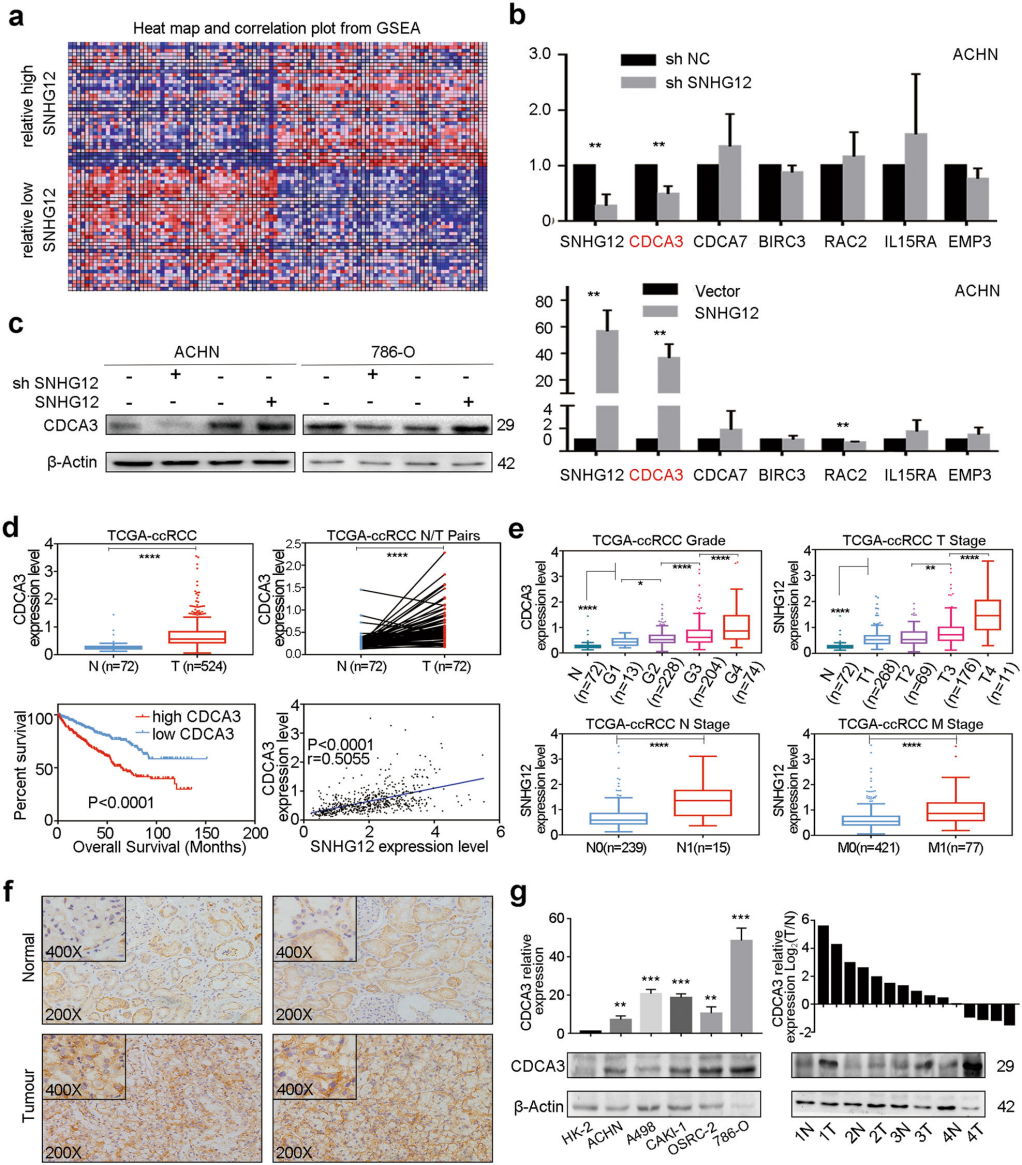
SNHG12 upregulated the expression of CDCA3 in RCC cells. **a** A heat map of correlated genes which were differentially expressed between relatively high SNHG12 expression and relatively low SNHG12 expression according to GSEA analysis based on data from GEO database (GSE53757). **b** The mRNA expression level of 6 candidate genes in ACHN cells with SNHG12 knockdown or overexpression. **c** The expression level of CDCA3 was verified by western blot analysis in transfected ACHN and 786-O cells. **d** Based on TCGA database, the mRNA level of CDCA3 was higher in ccRCC tissues than in normal tissues in TCGA database and higher CDCA3 expression indicated poorer prognosis in RCC. And CDCA3 expression level was positive correlated with SNHG12 expression (*r* = 0.5055, *P* < 0.0001). **e** The high expression of CDCA3 mRNA was related with various clinicopathological factors: G grade, T stage, lymph node metastasis and distant metastases. **f** Immunohistochemistry (IHC) for CDCA3 in ccRCC tissues and paired normal tissues **g** The mRNA and protein levels of CDCA3 in five RCC cell lines (786-O, A498, ACHN, CAKI-1, and OSRC-2) and normal cell line (293T). The mRNA and protein levels of CDCA3 in 20 RCC tissues and paired normal tissues. *P < 0.05, **P < 0.01, ***P < 0.001, ****P < 0.0001. Error bars indicate mean ± SD.

To investigate the potential mechanism of SNHG12 promoting the progression of RCC, GSEA analysis was performed based on data from GEO database (GSE53757^22^). As shown in Supplementary Fig. 3a–f, high expression of SNHG12 was related to many signalling pathways that are clearly involved in tumourigenesis and metastasis, such as the cell cycle checkpoint, cell migration, drug resistance, and epithelial-mesenchymal transition. We also accessed top 100 genes that related with SNHG12 by GSEA analysis, which was shown in the heatmap (Fig. 3a). With the intersection of these two parts, 6 tumour-related genes were selected (Supplementary Table 1). Then, the mRNA expression level of the 6 genes was detected by qRT-PCR after knocking down or overexpressing SNHG12 in 786-O or ACHN cells. Analysis of these six genes demonstrated that only the CDCA3 mRNA expression level changed similar to SNHG12 (Fig. 3b and Supplementary Fig. 3g, h). Western blotting showed that at the protein level, SNHG12 could regulate CDCA3 expression (Fig. 3c). In other words, the CDCA3 expression level might be positively regulated by SNHG12.

**Fig. 4:**
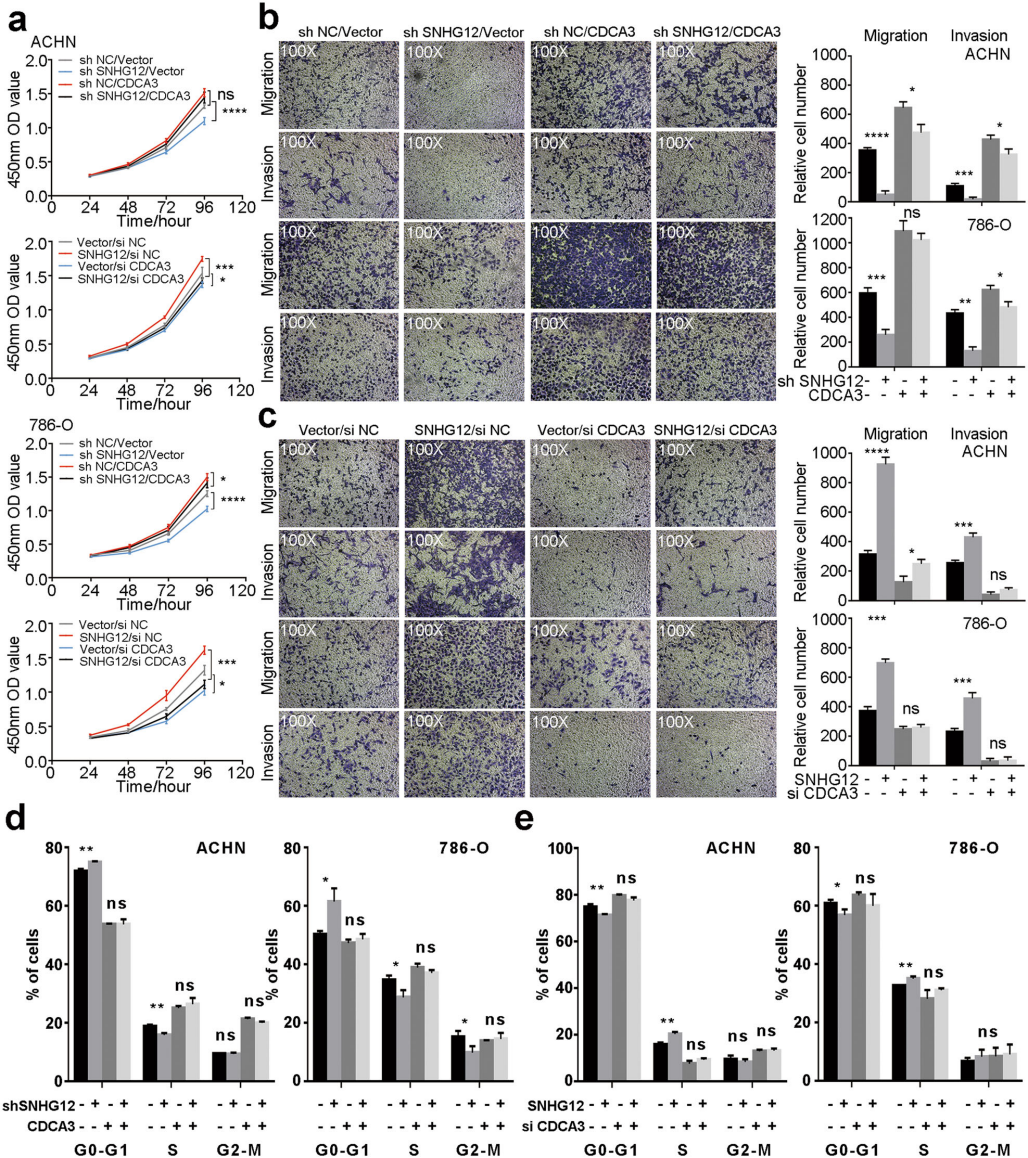
SNHG12 promoted tumour progression via CDCA3 in RCC cells. **a** CCK-8 assays showed overexpressing CDCA3 reversed the proliferation-inhibition effect of si SNHG12 and knockdown CDCA3 reversed the proliferation-promoting effect of SNHG12 overexpression in ACHN and 786-O cells. **b** Migration and invasion ability attenuated by sh SNHG12 could be reversed by CDCA3 overexpression in ACHN and 786-O cells according to transwell assays (magnification: ×100). **c** Migration and invasion ability promoted by SNHG12 could be reversed by si CDCA3 according to transwell assays (magnification: ×100). **d, e** Cell cycle distribution was analysed by PI staining in ACHN and 786-O cells after co-transfection by sh SNHG12 and CDCA3 overexpression vector or SNHG12 overexpression vector and si CDCA3 for 48 h. *P < 0.05, **P < 0.01, ***P < 0.001, ****P < 0.0001. Error bars indicate mean ± SD.

CDCA3, cell division cycle associated 3, has been reported to participate in numerous tumours, including gastric cancer^23^, colorectal cancer^24^, prostate cancer^25^ and so on. However, no study has focused on the role of CDCA3 in RCC yet. Thus, we analysed CDCA3 expression differences between RCC tissues and normal tissues in the TCGA-KIRC database. We found that CDCA3 expression was significantly higher in RCC tissues than in normal tissues, and a lower CDCA3 expression level indicated a better prognosis. Correlation analysis between CDCA3 and SNHG12 was performed, and they were found to be significantly positively correlated. Moreover, CDCA3 expression was highly related to numerous clinicopathological characteristics in ccRCC (Fig. 3d, e). Next, immunohistochemistry (IHC), qRT-PCR and western blotting were performed to further verify that CDCA3 was highly expressed in RCC tissues at both the mRNA and protein levels (Fig. 4f, g). Furthermore, after knocking down or overexpressing CDCA3 (Supplementary Fig. 4a), we studied the biological functions of CDCA3 in RCC cells. According to the results of CCK8 assays, transwell assays and cell cycle analysis, we found that CDCA3 had the capacity to enhance the proliferation, invasion and migration of RCC cells (Supplementary Fig. 4b–f).

### SNHG12 promoted tumour progression via CDCA3 in RCC cells

Here, rescue assays were performed to confirm the roles of the SNHG12/CDCA3 axis in RCC. As described above, 786-O and ACHN cells with SNHG12 stably knocked down or overexpressed were constructed by lentivirus infection. Then, CDCA3 was overexpressed by plasmid in sh SNHG12- and sh NC-treated RCC cells. In SNHG12-overexpressing cells and NC cells, CDCA3 expression was low due to CDCA3 siRNA. CCK8 assays showed that CDCA3 overexpression could rescue the negative effect of low SNHG12 expression on cell proliferation in RCC cells. Moreover, CDCA3 siRNA reversed the promotion of SNHG12 on RCC cell proliferation (Fig. 4a). Transwell assays were performed to assess cell migration and invasion. As shown in Fig. 4b, c, the migration and invasion of RCC cells inhibited by sh SNHG12 could be recovered through overexpression of CDCA3. Knocking down CDCA3 could also weaken the positive effect of SNHG12 on RCC cell migration and invasion. For cell cycle assays, we obtained similar results (Fig. 4d, e and Supplementary Fig. 5a, b). Overall, we found that SNHG12 promoted tumour progression via CDCA3 in RCC cells.

### SNHG12 increased sunitinib resistance in RCC cells through CDCA3

**Fig. 5:**
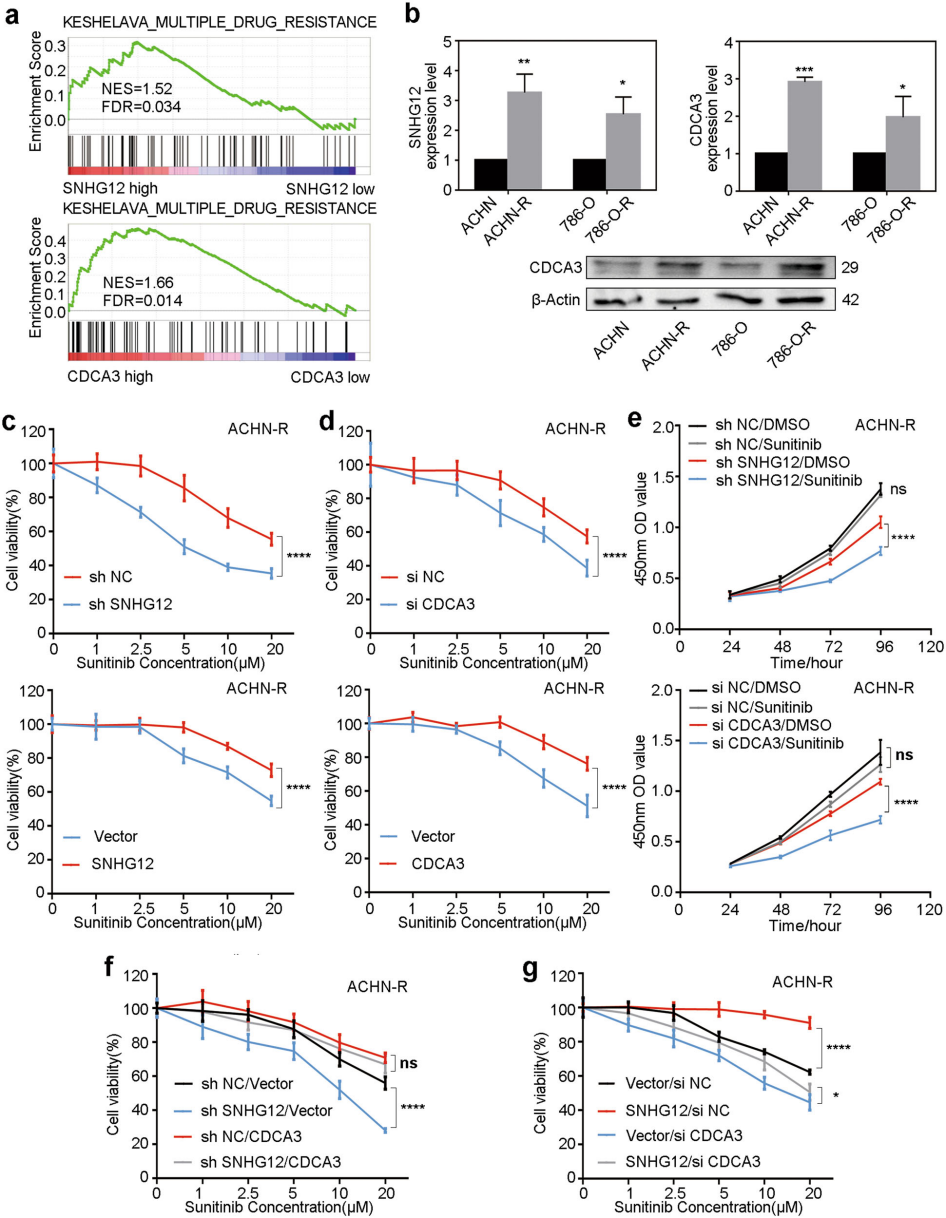
SNHG12 increased sunitinib resistance in RCC cells through CDCA3. **a** GSEA analysis for the correlation of multiple drug resistance and expression level of SNHG12 and CDCA3 according to GEO database (GSE53757. FDR < 0. 25, *P* < 0.05 was considered statistically significant). **b** qRT-PCR was used to determine the expression level of SNHG12 and CDCA3 in sunitinib-resistant RCC cells (ACHN-R and 786-O-R) and in RCC cells (ACHN and 786-O). And CDCA3 protein expression level was detected by western blot assays. **c** CCK-8 assays were used to measure sunitinib sensitivity of ACHN-R cells after transfection by sh SNHG12 or SNHG12 overexpression vector for 48 h. **d** CCK-8 assays were use**d** to measure sunitinib sensitivity of ACHN-R cells after transfection by si CDCA3 or CDCA3 overexpression vector for 48 h. **e** In ACHN-R cells, knocking down SNHG12 or CDCA3 revered sunitinib resistance, as described by CCK-8 assays. **f** The sensitivities o**f** ACHN-R after co-transfection by sh SNHG12 and CDCA3 overexpression vector under different sunitinib concentration were determined by CCK-8 assay. **g** The sensitivities of ACHN-R after co-transfection by SNHG12 overexpression vector and si CDCA3 under different sunitinib concentration were determined by CCK-8 assay. *P < 0.05, **P < 0.01, ***P < 0.001, ****P < 0.0001. Error bars indicate mean ± SD.

The differential expression analysis of lncRNA from GSE64052 already showed that SNHG12 was highly expressed in sunitinib-resistant RCC cells (Supplementary Fig. 6a). The TCGA analysis also revealed that both SNHG12 and CDCA3 were associated with multidrug resistance (Fig. 5a). Moreover, the PI3K/AKT pathway, a sunitinib-resistant related pathway^14,26^, was correlated with high expression of SNHG12 and CDCA3 (Supplementary Fig. 6b). Therefore, we hypothesised that the SNHG12/CDCA3 axis could play a role in sunitinib resistance. In a previous study, we established two sunitinib-resistant RCC cell lines, 786-O-R and ACHN-R^27^. First, qRT-PCR and western blotting assays were conducted to verify that SNHG12 and CDCA3 expression levels were higher in sunitinib-resistant cells (Fig. 5b). Then, the sunitinib sensitivity of ACHN-R was evaluated after SNHG12 knockdown or overexpression. It is clear that SNHG12 expression levels were negatively correlated with drug sensitivity in ACHN-R cells (Fig. 5c). Similarly, we also found that high expression of CDCA3 promoted ACHN-R cell sunitinib resistance (Fig. 5d). As shown in CCK8 assays, sunitinib-resistant RCC cell viability was not affected by 2.5 µM concentration of sunitinib when compared with DMSO. However, when SNHG12 or CDCA3 was knocked down, cell viability decreased in the presence of sunitinib (Fig. 5e and Supplementary Fig. 6c). These results revealed that knocking down SNHG12 or CDCA3 might increase the sensitivity of drug-resistant RCC to sunitinib. Nevertheless, under conditions of low SNHG12 expression, overexpressing CDCA3 with plasmid restored sunitinib resistance (Fig. 5f and Supplementary Fig. 6d). Similarly, in SNHG12-overexpressing cells, when CDCA3 was knocked down with siRNA, the enhanced drug tolerance could be reversed (Fig. 5g and Supplementary Fig. 6e). From these findings, we concluded that SNHG12 increased sunitinib resistance in RCC cells through CDCA3.

### SNHG12 bound to and stabilised SP1, which activated CDCA3 transcription

According to published articles, the transcription factors SP1 and HOXB3 could bind to the promoter sequence of CDCA3^23,25^. Studies have reported that nucleus-located lncRNAs could regulate protein transcription by binding to transcription factors^28,29^. To assess the possibility of SNHG12 interacting with these transcription factors, the *catRAPID* algorithm was used. Interestingly, the interaction strength between SNHG12 and SP1 was relatively higher, and potential binding sequences were predicted (Supplementary Fig. 7a, b). Thus, we mainly focused on SP1.

**Fig. 6:**
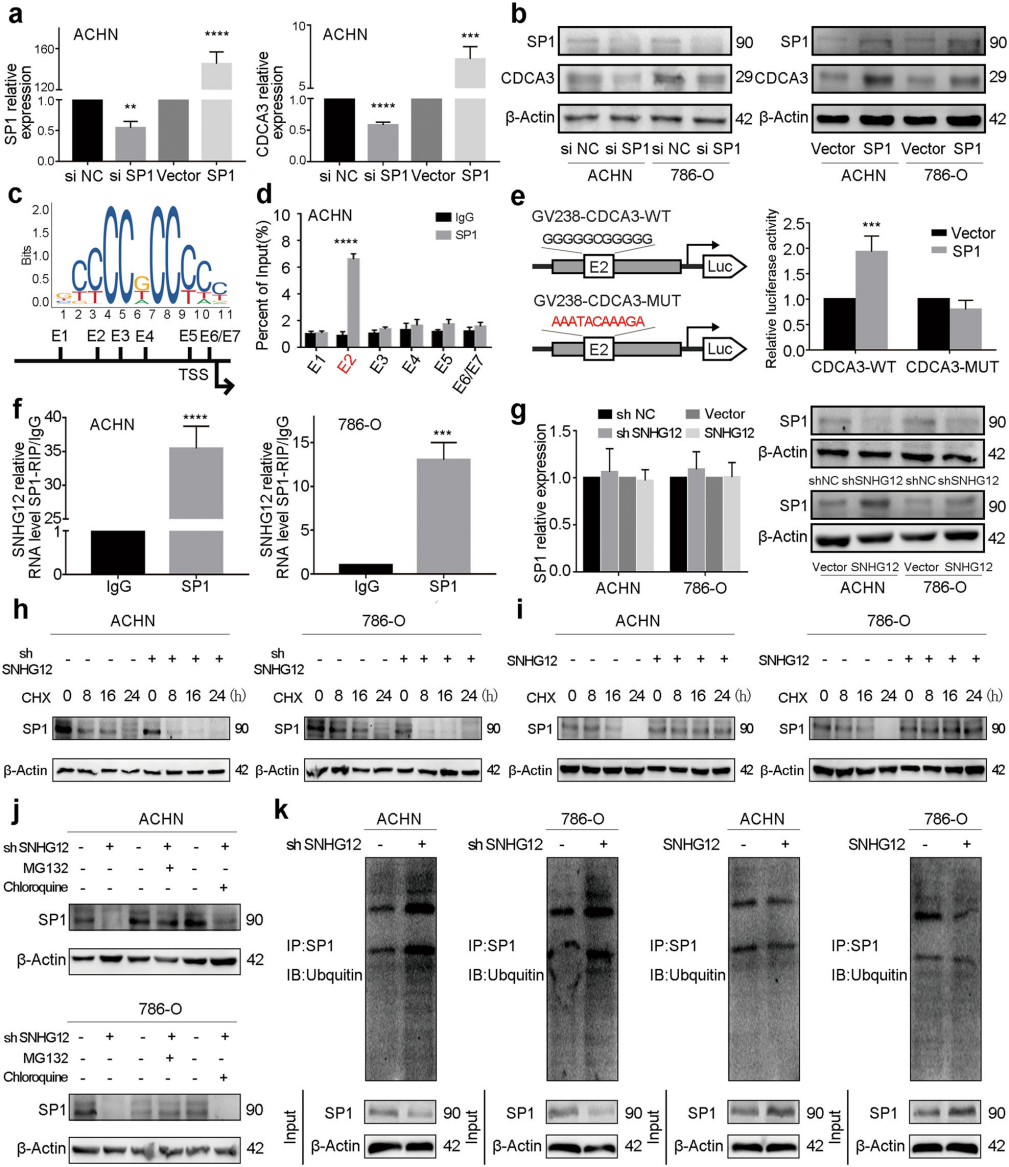
SNHG12 bound to and stabilised SP1, which activated CDCA3 transcription. **a** qRT-PCR for mRNA levels of SP1 and CDCA3 in transfected ACHN cells. **b** western blot assays for protein levels of SP1 and CDCA3 in transfected ACHN and 786-O cells. **c** The predicted positions of putative SP1 binding motif in −2000-bp human CDCA3 promoter. **d** ChIP-PCR assays were performed to show direct binding of SP1 to CDCA3 promoter regions in ACHN cells. **e** Luciferase reporter assays were performed by co-transfecting the wild type CDCA3 promoter or fragment E2-mutant CDCA3 promoter with SP1 overexpression vector or blank vector in 293T cells. **f** Anti-SP1 RIP-PCR assays were performed in ACHN and 786-O cells to show SP1 directly bound to SNHG12. **g** qRT-PCR and western blot for mRNA and protein levels of SP1 in transfected RCC cells. **h, i** SP1 protein levels were measured by western blot in RCC cells after transfected sh SNHG12 or SNHG12 overexpression vector and treated with cycloheximide (CHX) for a certain period of time. **j** Cells with SNHG12 knockdown were treated with vehicle (DMSO), MG132 (20 nM) or chloroquine (50 nM) for 24 h. Western blot assays were applied to show SP1 protein levels. **k** Immunoprecipitation with an anti-SP1 antibody were performed in SNHG12 knockdown or overexpression RCC cells, and analysed by western blotting with an anti-ubiquitin antibody. *P < 0.05, **P < 0.01, ***P < 0.001, ****P < 0.0001. Error bars indicate mean ± SD.

Next, we confirmed the expression promoting effect of SP1 on CDCA3 in RCC cells at the mRNA and protein levels (Fig. 6a, b and Supplementary Fig. 7c). Encouraged by this observation, we predicted the binding sites of SP1 in the CDCA3 promoter with JASPAR (Fig. 6c), and seven potential positions were identified. To validate the exact sites, a chromatin immunoprecipitation (ChIP) assay was performed. In both 786-O and ACHN cells, a strong enrichment between position E2 and anti-SP1 antibody was observed (Fig. 6d and Supplementary Fig. 7d). Furthermore, we constructed a CDCA3 promoter E2-wild-type (WT) GV238 vector and a CDCA3 promoter E2-mutant (MUT) GV238 vector. Luciferase activity analysis showed that the luciferase activity of the vector containing the WT CDCA3 promoter could be promoted by SP1 overexpression in 293T cells (Fig. 6e).

In addition, RNA-binding protein immunoprecipitation (RIP) assays were conducted to validate the interaction between SNHG12 and SP1. As shown in Fig. 6f, SNHG12 was significantly enriched by anti-SP1 antibody in 786-O and ACHN cells. However, when SNHG12 expression was low or high, the SP1 expression level showed inconsistent results at the mRNA and protein levels (Fig. 6g). Some scientists have demonstrated that lncRNAs play a role in post-translational regulation by affecting protein stability^28,30^. Based on this mechanism, cycloheximide (CHX), a protein synthesis inhibitor, was used to investigate whether SNHG12 regulates SP1 stability. Cell protein was isolated from SNHG12 knockdown and negative control RCC cells after CHX treatment for 0 h, 8 h, 16 h and 24 h. We found that the protein stability decreased when SNHG12 expression was relatively low (Fig. 6h). Moreover, when SNHG12 was overexpressed, SP1 tended to be more stable (Fig. 6i). As we know, there are different mechanisms of protein degradation, such as the ubiquitin/proteasomal-mediated pathway and autophagy/lysosomal-mediated pathway^31^. MG132, a proteasome inhibitor, and chloroquine, a lysosome inhibitor, were used to confirm the specific pathway involved in the SP1 stability regulated by SNHG12. As shown in Fig. 6j, when MG132 was added, SP1 remained stable even if SNHG12 was knocked down in RCC cells. Moreover, GO enrichment analysis and GSEA analysis revealed that SNHG12 was involved in numerous ubiquitin-related pathways (Supplementary Fig. 7e). Then, ubiquitination-related immunoprecipitation directly showed that the ubiquitination level of SP1 was regulated by SNHG12 in RCC cells (Fig. 6k). In conclusion, we determined that SNHG12 bound to and stabilised SP1, which activated CDCA3 transcription.

### SNHG12 knockdown repressed tumour progression and reversed sunitinib resistance in vivo

**Fig. 7:**
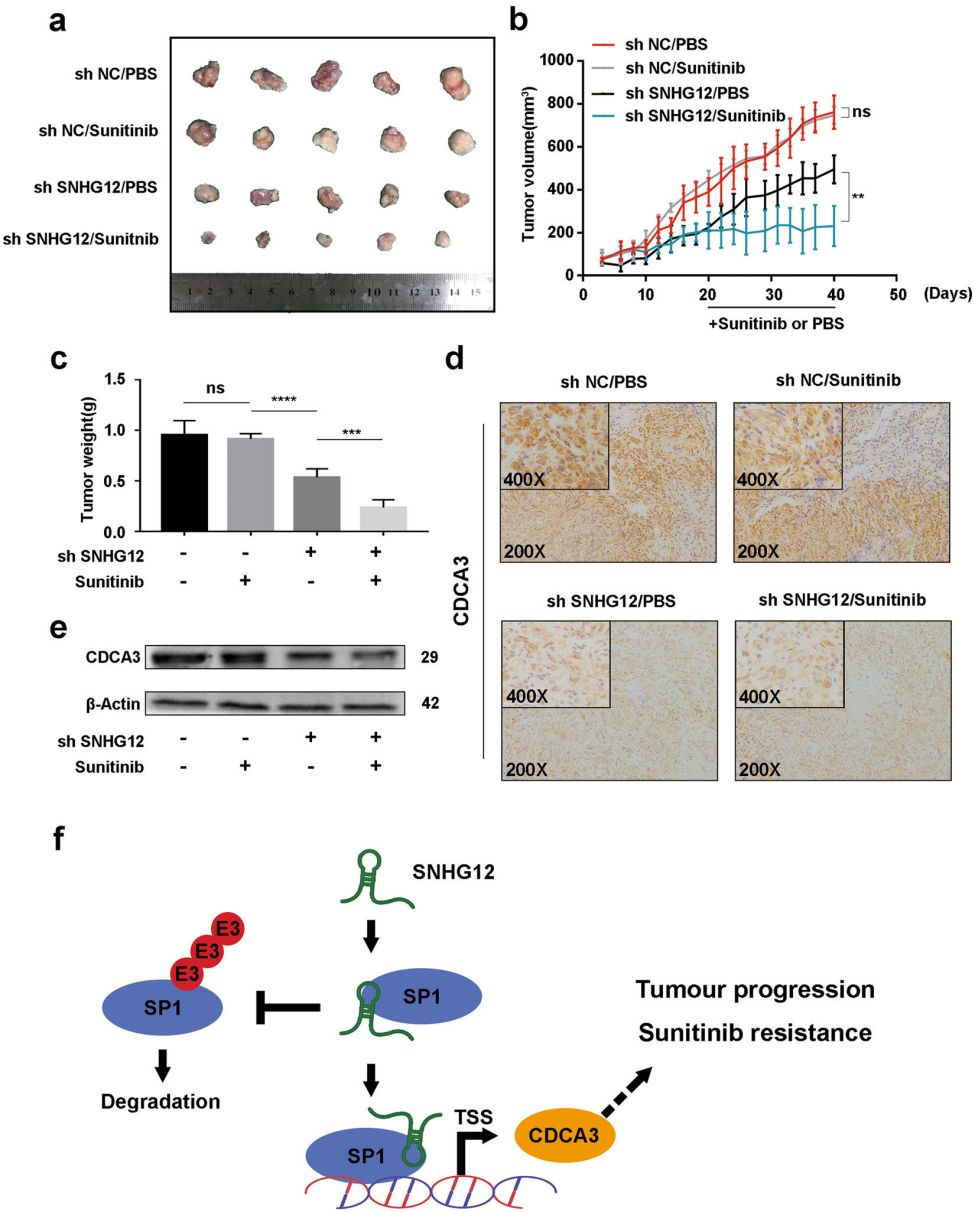
SNHG12 knockdown repressed tumour progression and reversed sunitinib resistance in vivo. **a** ACHN-R cells stably knockdown of SNHG12 were subcutaneously injected into nude mice. **b**, **c** The volume and weight of xenograft tumours in four groups with different treatment (*n* = 5). **d, e** Immunohistochemistry (IHC) and western blot assays were performed to measure CDCA3 expression levels in four groups with different treatment. **f** Schematic diagram illustrated lncRNA SNHG12 regulated SNHG12/SP1/CDCA3 axis promoting tumour progression and sunitinib resistance in RCC. *P < 0.05, **P < 0.01, ***P < 0.001, ****P < 0.0001. Error bars indicate mean ± SD.

Based on the results above, we studied the function of SNHG12 in vivo. A total of 3 × 10^6^ ACHN-R cells with SNHG12 stable knockdown were implanted subcutaneously into 10 4-week-old nude mice, while the same amount of negative control ACHN-R cells were implanted into 10 other nude mice. From day 20, sunitinib or vehicle was fed to five mice of each group every day. Tumour size was measured every 2 or 3 days starting with tumour formation to day 40. From the results, we observed that knocking down SNHG12 inhibited tumour growth (Fig. 7a). Moreover, in the sh SNHG12 group, both the volume and weight of tumours from mice fed sunitinib were obviously decreased, which revealed that low SNHG12 expression increased RCC sensitivity to sunitinib (Fig. 7a–c). IHC and western blotting were performed in these xenograft tumours. We found that in the sh SNHG12 group, CDCA3 was expressed at low levels (Fig. 7d, e).

In Fig. 7f, we generated a schematic diagram of this process. SNHG12 could bind to SP1 and inhibit its ubiquitination and degradation in RCC. In addition, stabilised SP1 promoted the expression of CDCA3, which resulted in RCC progression and sunitinib resistance.

## Discussion

In the present study, we demonstrated that lncRNA SNHG12 plays an essential role in RCC. First, by RNA sequencing of RCC tissues and bioinformatics analysis, we found that SNHG12 expression was higher in RCC tissues and in sunitinib-resistant RCC cells. The higher expression level of SNHG12 was also linked with higher tumour grade and indicated a worse prognosis. Next, functional experiments showed that SNHG12 could promote RCC cell proliferation, migration, invasion and sunitinib resistance. Then, we verified that SNHG12 could regulate the expression of CDCA3 by GSEA analysis and qRT-PCR assays. Furthermore, the SNHG12/CDCA3 axis could promote RCC progression and sunitinib resistance in vitro and in vivo. Mechanically, SNHG12 bound to the transcription factor SP1, which reduced SP1 ubiquitination. The protein stability of SP1 was increased, and as a result, CDCA3 was highly expressed.

Recently, increasing studies have discovered the underlying mechanisms of long noncoding RNAs at multiple regulatory levels^32^. For SNHG12, almost all existing studies agreed that it works as a miRNA sponge in different cancer cells^16,18,33,34^. By this mechanism, SNHG12 functions as an endogenous competitive mimic target of miRNAs, which results in the upregulation of the mRNA that is regulated by these miRNAs. However, in most of these studies, the location of SNHG12 or target miRNA were not discussed, and the target miRNAs were mainly predicted from several specific databases. In our study, a cellular fractionation assay of RNA and fluorescence in situ hybridisation (FISH) assays were performed, and we confirmed that SNHG12 is mainly located in the nucleus. This result was consistent with the findings from Sun et al.^35^. On the basis of this result, we assumed that SNHG12 could play a regulatory role at the transcriptional level in the nucleus. Previously, Jiang et al. demonstrated that SNHG15, a member of the SNHG family, interacted with and stabilised the transcription factor Slug and promoted colon cancer progression^28^. Post-translational modification is an important and common regulatory mechanism in proteomics, and lncRNAs are involved in various types of comprehensive modifications, such as phosphorylation^36^, ubiquitination^37^ and acetylation^38^. The SP1 transcription factor, an essential transcription factor, is involved in many cellular processes. It could be activated or repressed by different post-translational modifications, including phosphorylation, ubiquitination and glycosylation^39^. Wang et al. clarified that RNF4 acted as the E3 ubiquitin ligase and initiated ubiquitin-mediated proteolysis of SP1^40^. In our study, RIP assays demonstrated that SNHG12 directly bound to SP1 in RCC cells. Subsequent protein stability assays showed that highly expressed SNHG12 stabilised the SP1 protein. Furthermore, we could clearly see by co-immunoprecipitation assay that more ubiquitin bound to SP1 when SNHG12 was knocked down. Hence, we found that SNHG12 could inhibit SP1 ubiquitin-mediated proteolysis in RCC cells, which was a completely different regulatory mechanism of SNHG12 from previous studies.

CDCA3 is a protein that has not been researched deeply. Published articles about CDCA3 mainly focused on the role of CDCA3 in cell cycle regulation and drug resistance in digestive tumours and lung cancer^24,41^. It has been reported that CDCA3 functions as an F-box protein that leads to the ubiquitylation-dependent proteolysis of Wee1 and ultimately results in irreversible mitotic entry^42^. In our research, we found for the first time that CDCA3 upregulated by SNHG12/SP1 was related to RCC and was involved in sunitinib resistance. SNHG12/SP1/CDCA3 might increase the cell cycle transition from G1 phase to S phase and promote cell proliferation in combination with sunitinib. However, the specific mechanism of SNHG12/SP1/CDCA3 regulating sunitinib resistance in RCC remains unclear and needs further studies.

There are limited therapeutic options for advanced metastatic RCC patients. With the use of receptor tyrosine kinase inhibitors (RTKIs), including sunitinib and sorafenib, the overall survival of these patients has been prolonged considerably. Unfortunately, the majority of patients develop sunitinib resistance after 6–15 months, which eventually leads to tumour progression^5–7^. Hence, it is urgent to discover potential mechanisms and identify biomarkers of sunitinib resistance. Studies have revealed that compensatory negative feedback in receptor tyrosine kinase pathways is an important mechanism of adaptive resistance to targeted therapy of cancer^43^. As the downstream effector of VEGFR, the PI3K/AKT signalling pathway was reported to be activated in drug-resistant cells^14^. Furthermore, a phase II study of combination therapy with perifosine, an inhibitor of AKT, and sorafenib in patients with lymphoproliferative diseases has already been completed^44^. In this study, we found that lncRNA SNHG12 was highly expressed in sunitinib-resistant RCC cells. SNHG12 attenuated sunitinib sensitivity by promoting CDCA3 expression via the transcription factor SP1. In addition, by GSEA analysis, we discovered that the PI3K/AKT signalling pathway was positively correlated with SNHG12 and CDCA3, which suggested that the SNHG12/SP1/CDCA3 axis might promote sunitinib resistance via the PI3K/AKT signalling pathway. However, more detailed studies are needed to explore the specific mechanism.

In summary, we demonstrated for the first time that long noncoding RNA SNHG12 could upregulate the expression of CDCA3 by stabilising the transcription factor SP1 and thereby promoting tumour progression and sunitinib resistance in RCC. Our findings in this study could provide a novel diagnostic and therapeutic target for RCC.

## Materials and methods

### Human RCC tissue samples and cell culture

A total of 50 matched RCC and adjacent normal renal tissues were obtained from the Department of Urology, Wuhan Union Hospital between September 2016 and September 2018. The present study was approved by the Ethics Committee of Human Research of Huazhong University of Science and Technology (HUST), and written informed consent was obtained from patients prior to surgery.Human RCC cell lines (786-O, ACHN, A498, OSRC-2 and Caki-1) and the human renal proximal tubular epithelial cell line HK-2 were purchased from the American Type Culture Collection (ATCC) in the USA. These cells were cultured in high-glucose DMEM containing 10% foetal bovine serum and 1% streptomycin-penicillin (Google Biotechnology, Wuhan, China) and maintained in a humidified atmosphere with 5% CO2 at 37 °C.

### Transient transfection and lentivirus infection assay

Short hairpin RNA (shRNA) against SNHG12 (sh SNHG12) and a negative control shRNA (sh NC) were designed and synthesised by Vigene (Vigene Biology, Shangdong, China). The small interfering RNAs (siRNAs) si CDCA3, si SP1 and si NC (cat. no. siN0000002‑1‑5) were obtained from Guangzhou RiboBio (RiboBio, Guangzhou, China). The RNA sequences used for transfection in this study are shown in Supplementary Table 2. In addition, the pcDNA3.1 vector (Vigene Biology) containing the full-length cDNA sequence of SNHG12, CDCA3 or SP1 was used to overexpress SNHG12, CDCA3 or SP1, respectively. The empty pcDNA3.1 vector was used as a negative control. For cell transient transfection, Lipofectamine 2000 reagent (Invitrogen, Carlsbad, CA) was used according to the manufacturer’s instructions when the RCC cell lines were at 70–80% confluence.

The lentivirus pLent-shSNHG12-GFP-Puro or its negative control (NC) pLent-GFP-Puro (Vigene Biology) was used to infect RCC cells with enhanced infection solution (Vigene Biology) according to the manufacturer’s protocol. Similarly, pLent-SNHG12-GFP-Puro lentivirus or empty vector (vector) pLent-GFP-Puro lentivirus (Vigene Biology) was used to overexpress genes. Seventy-two hours after the cells were infected with lentivirus, 2 μg/ml puromycin was added to kill the cells that had not been transfected.

### Quantitative reverse transcription PCR

Total RNA was isolated from tissues or cells using TRIzol Reagent (Thermo Fisher Scientific, Massachusetts, USA). Then, the extracted RNA was reverse transcribed into cDNA using a Superscript II reverse transcription kit (Takara Bio, Beijing, China) according to the manufacturer’s protocols. Subsequently, qRT-PCR was conducted with a SYBR-Green master kit (Vazyme, Nanjing, China) on a LightCycler 480 II (Roche Diagnostics) instrument according to the manufacturer’s protocols. The primers used to amplify SNHG12, CDCA3, CDCA7, BIRC3, RAC2, IL15RA, EMP3, SP1 and GAPDH were chemically synthesised by TSINGKE (TSINGKE, Beijing, China). All qRT‑PCR reactions were performed in triplicate. The primer sequences used for qRT-PCR in this study are shown in Supplementary Table 3.

### Cellular fractionation assay of RNA

The cellular fraction assay was performed using a PARIS™ Kit (Invitrogen, USA) according to the manufacturer’s instructions. GAPDH mRNA was used as a cytoplasmic control, and U6 RNA was used as a nuclear control. The fractionated RNA was examined by qRT PCR in triplicate.

### RNA fluorescence in situ hybridisation

Cy3-labelled SNHG12 probes as well as Cy3-labelled U6 and 18S probes used as positive controls for the nucleus and cytoplasm, respectively, were purchased from RiboBio. Hybridisation was performed with a Fluorescent in Situ Hybridization kit (RiboBio) according to the manufacturer’s instructions. Slides were observed using an IX71 inverted microscope (Olympus, Japan). RNA FISH experiments were performed in the 786-O and ACHN cell lines.

### Western blotting

Proteins from tissues and cells were extracted using RIPA buffer (Servicebio, Wuhan, China) containing protease inhibitors. Then, protein concentrations were determined using a BCA Protein Assay kit (Beyotime Institute of Biotechnology). A total of 30 μg of protein was subjected to 10% SDS-PAGE and transferred to a polyvinylidene difluoride (PVDF) membrane (EMD Millipore, Bedford, USA). PVDF membranes were then blocked in 5% skim milk for 2 h. Subsequently, samples were incubated with specific primary antibodies against CDCA3, SP1, ubiquitin and β-actin at 4 °C overnight. Next, membranes were incubated with the appropriate secondary antibodies for 2 h at room temperature. Finally, the protein bands were visualised with Pierce™ ECL Western Blotting Substrate (Thermo Fisher Scientific) using ChemiDoc XRS+(Bio‑Rad Laboratories, Inc.). Information on the antibodies is shown in Supplementary Table 4.

### Co-immunoprecipitation

Forty-eight hours after transfection, the cells were lysed in Triton lysis buffer. After that, 20 µL of protein A/G PLUS-Agarose (Santa Cruz, CA, USA) together with anti-SP1 antibody or control IgG antibody were added to the supernatant of centrifuged lysates and incubated overnight at 4 °C. The next day, protein A/G PLUS-Agarose was precipitated and washed with Triton lysis buffer three times. Finally, 2X SDS sample buffer was added to the immunoprecipitates, and the samples were subjected to western blotting after boiling for 10 min.

### Chromatin immunoprecipitation

ChIP assays were conducted following the protocol of the EZ-ChIP™ kit (Millipore, Billerica, MA). Chromatin was sonicated to obtain 150–900-bp DNA/protein fragments after cells were cross-linked with formaldehyde. Then, anti-SP1 or control antibody was added to lysates and incubated at 4 °C overnight. The next day, ChIP-Grade Protein G Agarose Beads were used for immunoprecipitation. After that, DNA was released and purified for subsequent qRT-PCR.

### RNA-binding protein immunoprecipitation (RIP)

RIP assays were performed following the protocol of the RIP™ RNA binding protein immunoprecipitation kit (Millipore). Cells were lysed with complete RIP lysis buffer. Then, anti-SP1 antibody or control IgG antibody was incubated with magnetic beads for 30 min. After that, the beads were added to lysates and incubated at 4 °C overnight. Finally, RNA was purified for subsequent qRT-PCR assay.

### Luciferase reporter assays

The sequence of the CDCA3 promoter sites (−2000 to transcription initiation site) containing the wild-type binding sequence or mutant type binding sequence was cloned in a GV238-based vector. 293T cells were transfected with the GV238-CDCA3-WT or GV238-CDCA3-MUT plasmid. After 24 h, cells were lysed, and luciferase activity was measured with a Dual-Luciferase Reporter Assay Kit (Promega, Madison, WI, USA). Renilla luciferase was used for normalisation. All transfections were repeated in triplicate.

### Immunohistochemistry assay

Briefly, tissues obtained from ccRCC patients or tumour xenograft mice were sequentially fixed in formalin at room temperature for 12 h, dehydrated and embedded in paraffin. Then, IHC was conducted with rabbit antibodies against CDCA3 (1:100, Abcam, Cambridge, UK). Finally, tissue slides were observed in three randomly selected fields under a light microscope (Olympus Corporation) at ×5 and ×200 magnification.

### Cell proliferation assay

The proliferation ability of ACHN and 786-O cells was assessed with the Cell Counting Kit-8 (Dojindo Molecular Technologies, Kyushu, Japan) reagent according to the manufacturer’s instructions. Cells were inoculated on 96-well plates at a density of 1 × 10^3^ cells per well with 100 μl of medium. Every 24 h for a total of 96 h, CCK8 solution (10 μl) was added to each well, and the cells were further incubated at 37 °C for 3 h. The absorbance of each well was measured at 450 nm with a spectrophotometer.

### Flow cytometry cell cycle assay

After transient transfection, ACHN and 786-O cells were fixed in 75% ethanol for 12 h. Then, cells were stained with propidium iodide (Beyotime) for cell cycle analysis. Finally, the percentage of cells in each cell cycle phase (G0/G1, S and G2/M) was assessed, and the results were analysed by ModFit LT software.

### Cell migration and invasion assays

Migration and invasion assays were performed as previously described^45^. After starving the cells for 6–8 h in serum-free DMEM, a total of 1 × 10^4^ cells were seeded in the upper chamber with 200 μl of serum-free medium for the migration assay. In addition, 2 × 10^4^ cells were added into Matrigel‑coated upper Transwell chambers for the invasion assay. The lower chambers were filled with DMEM containing 10% FBS. After incubation at 37 °C for 24 h, cells on the lower surface of the membrane were fixed in 100% methanol and stained with 0.1% crystal violet dye for 20 min at room temperature. Finally, after washing with PBS, cells were imaged in five randomly selected fields under a light microscope (Olympus Corporation) at ×100 magnification.

### Tumour xenograft model

Four-week-old male BALB/c nude mice (HFK Bio-technology, Beijing, China) were chosen for the tumour xenograft model. A total of 3 × 10^6^ ACHN-R cells stably transfected with sh SNHG12 or sh NC were subcutaneously injected into the upper back of each mouse (*n* = 10 mice/group). Tumour sizes were measured every 2 or 3 days. After 20 days, 5 mice in the sh SNHG12 group and 5 mice in the sh NC group were fed sunitinib, while the remaining mice in the two groups were fed vehicle. Mice were killed after 40 days. Tumour size was measured, and IHC assays were conducted. The animal experiments were approved by the Animal Ethics Committee of HUST.

### Bioinformatics analysis

SNHG12 expression levels in ccRCC specimens and correlated clinical data were downloaded from The Cancer Genome Atlas database (TCGA; https://xenabrowser.net/heatmap/), GEO database (GSE64052, GSE53757; https://www.ncbi.nlm.nih.gov/geo/) and Oncomine database (Yusenko Renal dataset; https://www.oncomine.org). The gene set enrichment analysis (GSEA; http://software.broadinstitute.org/gsea/index.jsp) was performed to determine signalling pathways and molecules involved in the pathogenesis of ccRCC when SNHG12 was highly expressed.

### Statistical analysis

In the present study, statistical analyses were performed by GraphPad Prism 7.0 (GraphPad Software, San Diego, California, USA) and SPSS 22.0 (IBM, NY, USA). Student’s *t* test or paired Student’s *t* test, receiver operator characteristic curve, Pearson *χ*^2^ test, Cox regression analysis, linear regression and Kaplan–Meier curve with log-rank test were conducted as indicated. Significance was determined at *P* < 0.05.

## Supplementary information

Supplementary Tables

Supplementary Figure 1

Supplementary Figure 2

Supplementary Figure 3

Supplementary Figure 4

Supplementary Figure 5

Supplementary Figure 6

Supplementary Figure 7

Supplementary Figure legends
