# Supplementary material for: Long noncoding RNA SNHG12 promotes tumour progression and sunitinib resistance by upregulating CDCA3 in renal cell carcinoma
Source: Cell Death Dis. 2020 Jul 8;11(7):515. doi: 10.1038/s41419-020-2713-8 (PMC7343829; doi:10.1038/s41419-020-2713-8)
Supplement: Supplementary file 1 — Supplementary Tables [file 41419_2020_2713_MOESM1_ESM.docx]

**Supplementary Table 1. The six candidate genes correlated with SNHG12.**

| **Entrez ID** | **Gene symbol** | **Signaling pathway** | **Pearson r** | **P value** |
| --- | --- | --- | --- | --- |
| 83461 | CDCA3 | Metastasis signaling pathway; EGFR signaling pathway | 0.5055 | <0.0001 |
| 83879 | CDCA7 | Epithelial mesenchymal translation signaling pathway | 0.4048 | <0.0001 |
| 330 | BIRC3 | Cell cycle signaling pathway | 0.4261 | <0.0001 |
| 5880 | RAC2 | Cell migration signaling pathway | 0.4204 | <0.0001 |
| 3601 | IL15RA | Cell apoptosis signaling pathway | 0.5163 | <0.0001 |
| 2014 | EMP3 | Cell migration signaling pathway | 0.4641 | <0.0001 |

**Supplementary Table 2. shRNA and siRNA sequence used in this study.**

| **Name** | **Sequence** | **Company** |
| --- | --- | --- |
| SNHG12-shRNA | GATCCGCTGTCCTCATTTGTGACTATTTCAAGAGAATAGTCACAAATGAGGACAGCTTTTTTA | Vigene Biology |
| CDCA3-siRNA | GTACCCAGTTATCTGTTGA | RiboBio |
| SP1-siRNA | GCCAATAGCTACTCAACTA | RiboBio |

**Supplementary Table 3. Primers sequence used in this study.**

| **Primers used for quantitative RT-PCR** | | |
| --- | --- | --- |
| **Name** | **Forward-primer** | **Reverse-primer** |
| SNHG12 | TCTGGTGATCGAGGACTTCC | ACCTCCTCAGTATCACACACT |
| BIRC3 | TGGCATCTGGTAACTTTTGACTG | TGGTAGACAACAGGTGCTGC |
| CDCA3 | TGGTATTGCACGGACACCTA | TGTTTCACCAGTGGGCTTG |
| CDCA7 | CGGCTTTTCAGAAAGTGAGG | CATCGTCAGAGTCGGCATGA |
| EMP3 | AAGACTCCGACTCCAGCTCT | CTGACATTACTGCAGGCCCA |
| IL15RA | CCAGCCGCCAGGTGTGTATC | GGTTTCCCCGAGTTTCATAGGTG |
| RAC2 | CGTCAGCCCAGCCTCTTATG | TCAGGCCTCTCTGGGTGAG |
| SP1 | CCTGGTCATACTGTGGGAAAC | GGTGGGTCTTGATATGTTTTGAC |
| GAPDH | AAAAGCATCACCCGGAGGAGAA | AAGGAAATGAATGGGCAGCCG |
| U6 | TGCGGGTGCTCGCTTCGGCAGC | CCAGTGCAGGGTCCGAGGT |
| **Primers used for ChIP-qPCR** | | |
| **Name** | **Forward-primer** | **Reverse-primer** |
| SP1 binding site 1 | TCAGGCCATGAGCTGGTTCT | CTATGTCACCCTCAAGCGGA |
| SP1 binding site 2 | CCTCCGAGAAAGAAACCACCT | TTTGAAGTGAGCAATGGCCGC |
| SP1 binding site 3 | ACGACTAGAGCTTTCCTCTCCT | CCTCCACCCCGTGGTTAAAA |
| SP1 binding site 4 | CGCCTAGGAACTGCGGTC | GGCTCTCCGGGTTTTGTAGT |
| SP1 binding site 5 | AATGACCAGGGCGATGGAAG | GAGACCGGGTCCACAAAGAC |
| SP1 binding site 6/7 | AATGAGCGCCACTCGTTAGG | CTCATTGGCGTCAGTCAAGC |
| control | GGACGGTAGTCACACGACAG | GGACTTGTAGTCTCCCACGC |

**Supplementary Table 4. Antibodies used in this study.**

| **Name** | **Company** | **Catalog Number** | **Assay** |
| --- | --- | --- | --- |
| CDCA3 | Abcam | #AB166902 | WB, IHC |
| SP1 | Cell Signaling Technology | #9389 | WB, IP, RIP,CHIP |
| Ubiquitin | Abcam | #AB7254 | WB |
| β-actin | Proteintech | #60008-1-Ig | WB |
| HRP-conjugated Affinipure Goat Anti-Mouse IgG(H+L) | Proteintech | #SA00001-1 | WB |
| HRP-conjugated Affinipure Goat Anti-Rabbit IgG(H+L) | Proteintech | # SA00001-2 | WB |
