# Supplementary figures and images for: Long noncoding RNA SNHG12 promotes tumour progression and sunitinib resistance by upregulating CDCA3 in renal cell carcinoma
Source: Cell Death Dis. 2020 Jul 8;11(7):515. doi: 10.1038/s41419-020-2713-8 (PMC7343829; doi:10.1038/s41419-020-2713-8)

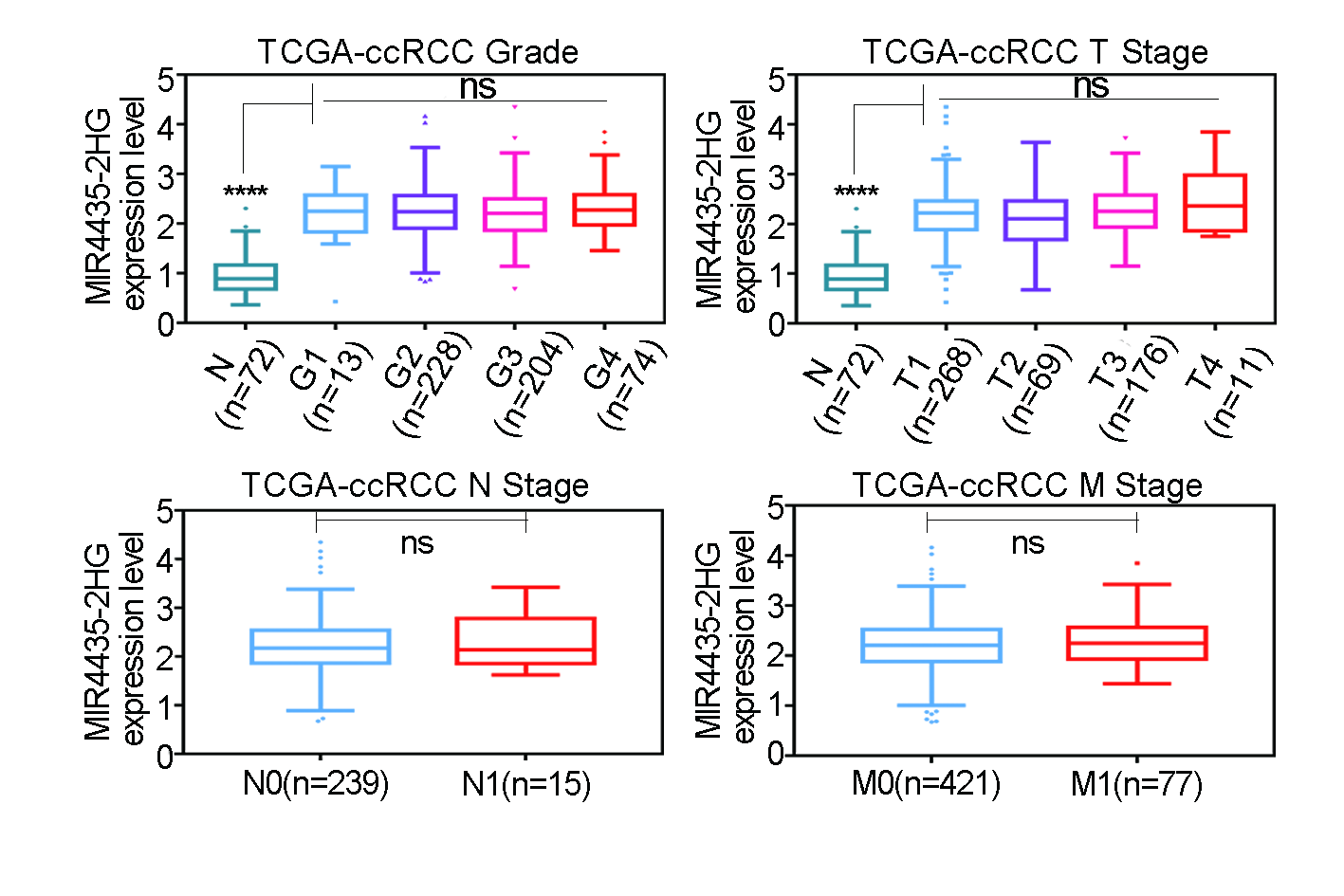

Supplement: Supplementary file 2 — Supplementary Figure 1 [file 41419_2020_2713_MOESM2_ESM.tif]

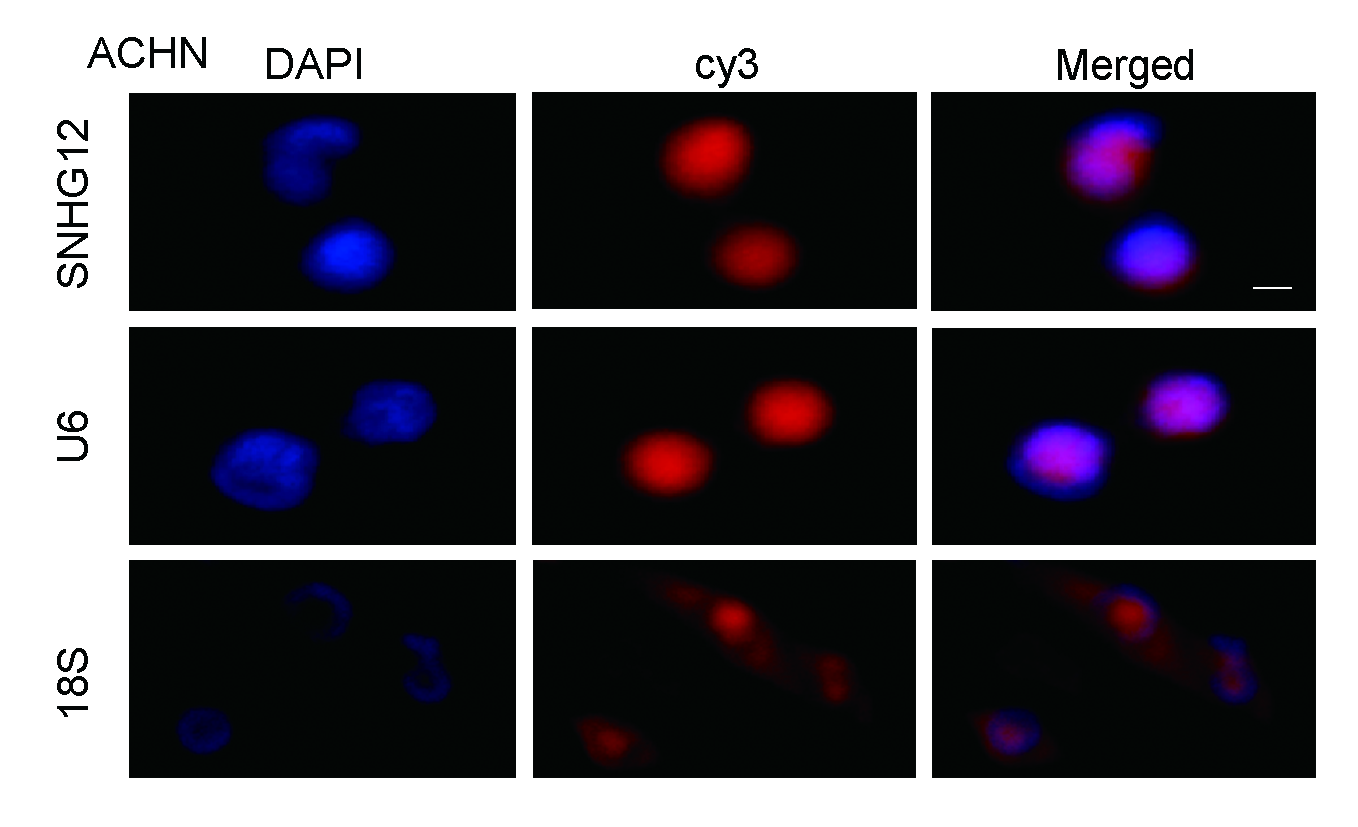

Supplement: Supplementary file 3 — Supplementary Figure 2 [file 41419_2020_2713_MOESM3_ESM.tif]

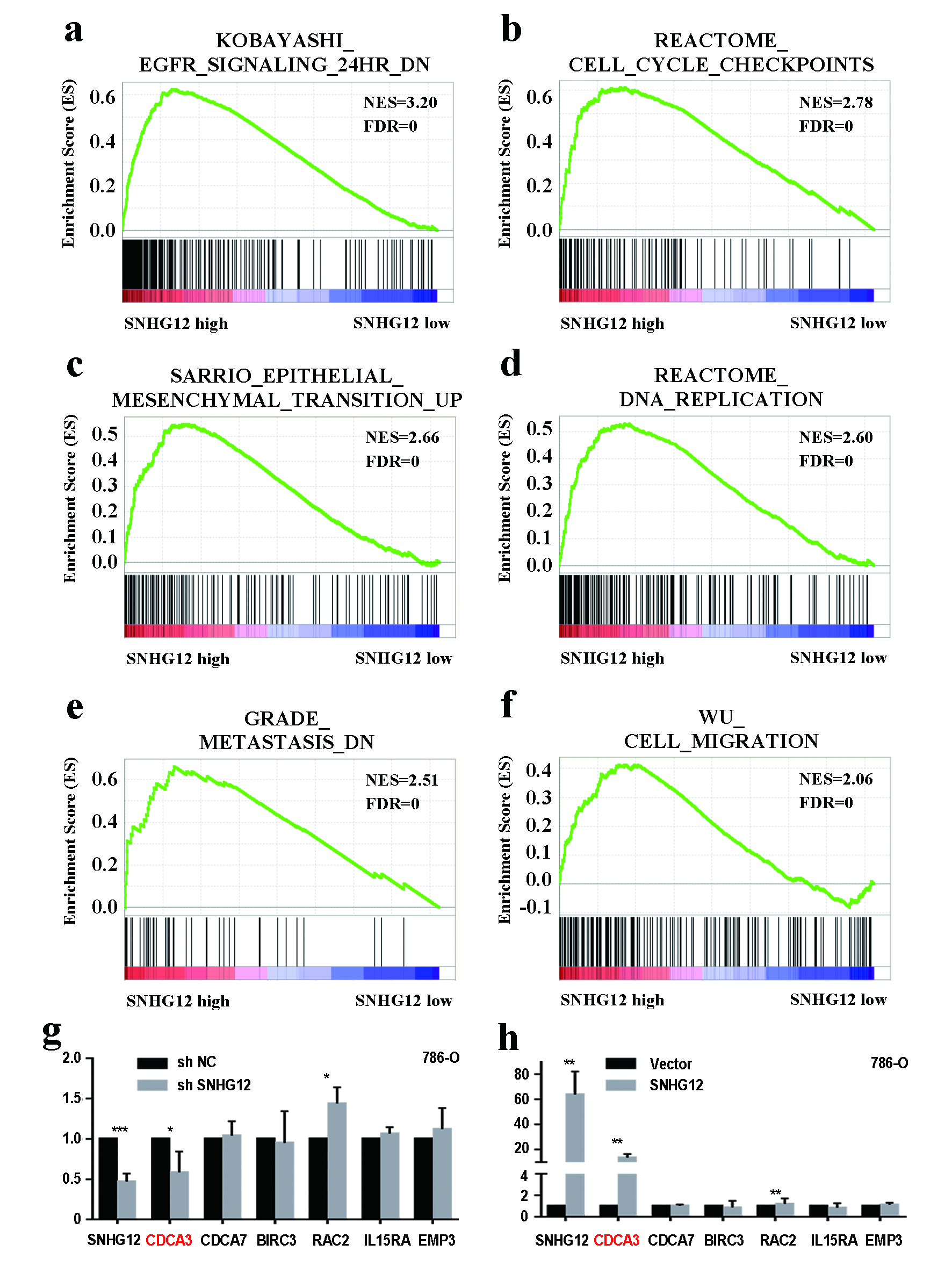

Supplement: Supplementary file 4 — Supplementary Figure 3 [file 41419_2020_2713_MOESM4_ESM.tif]

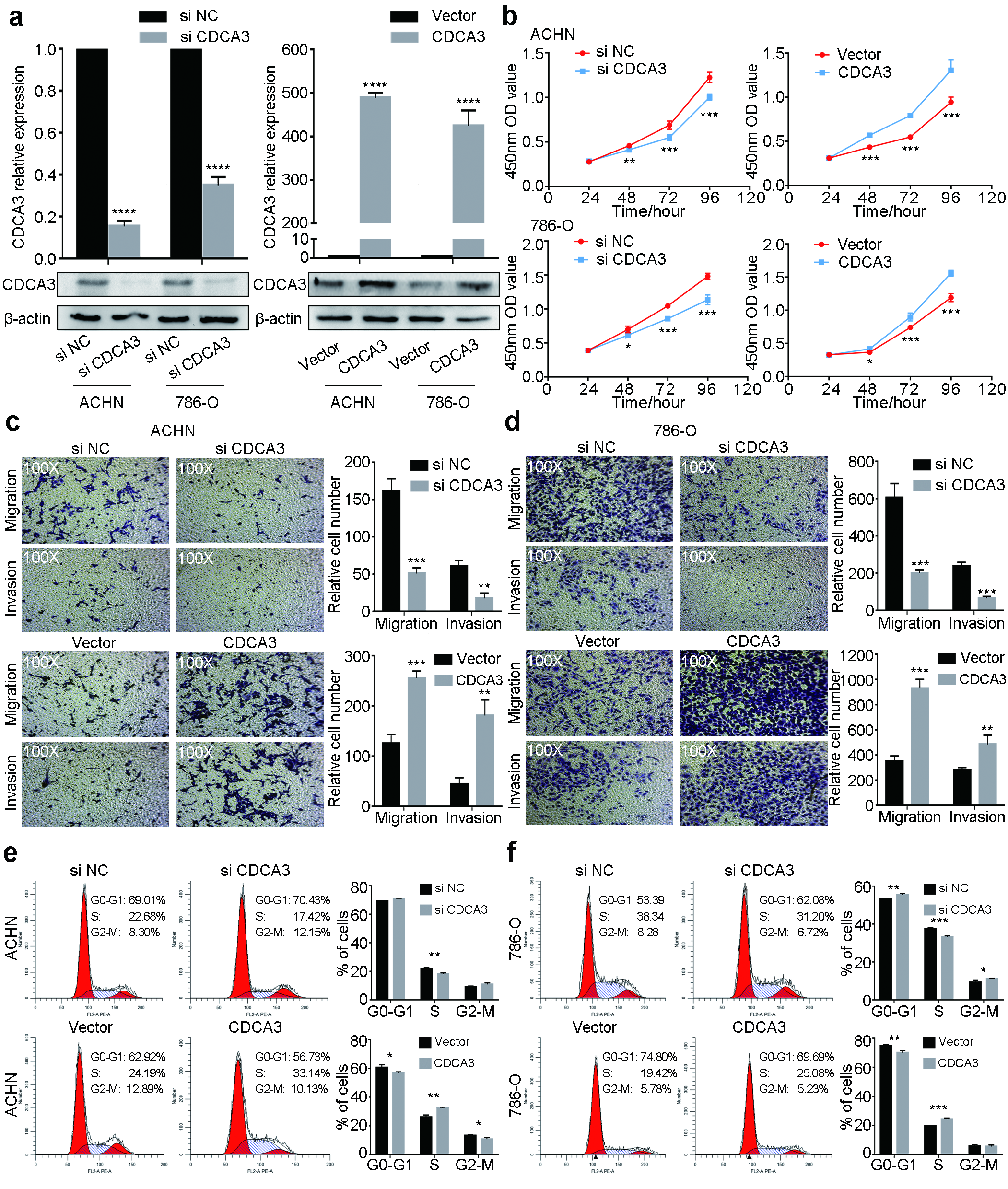

Supplement: Supplementary file 5 — Supplementary Figure 4 [file 41419_2020_2713_MOESM5_ESM.tif]

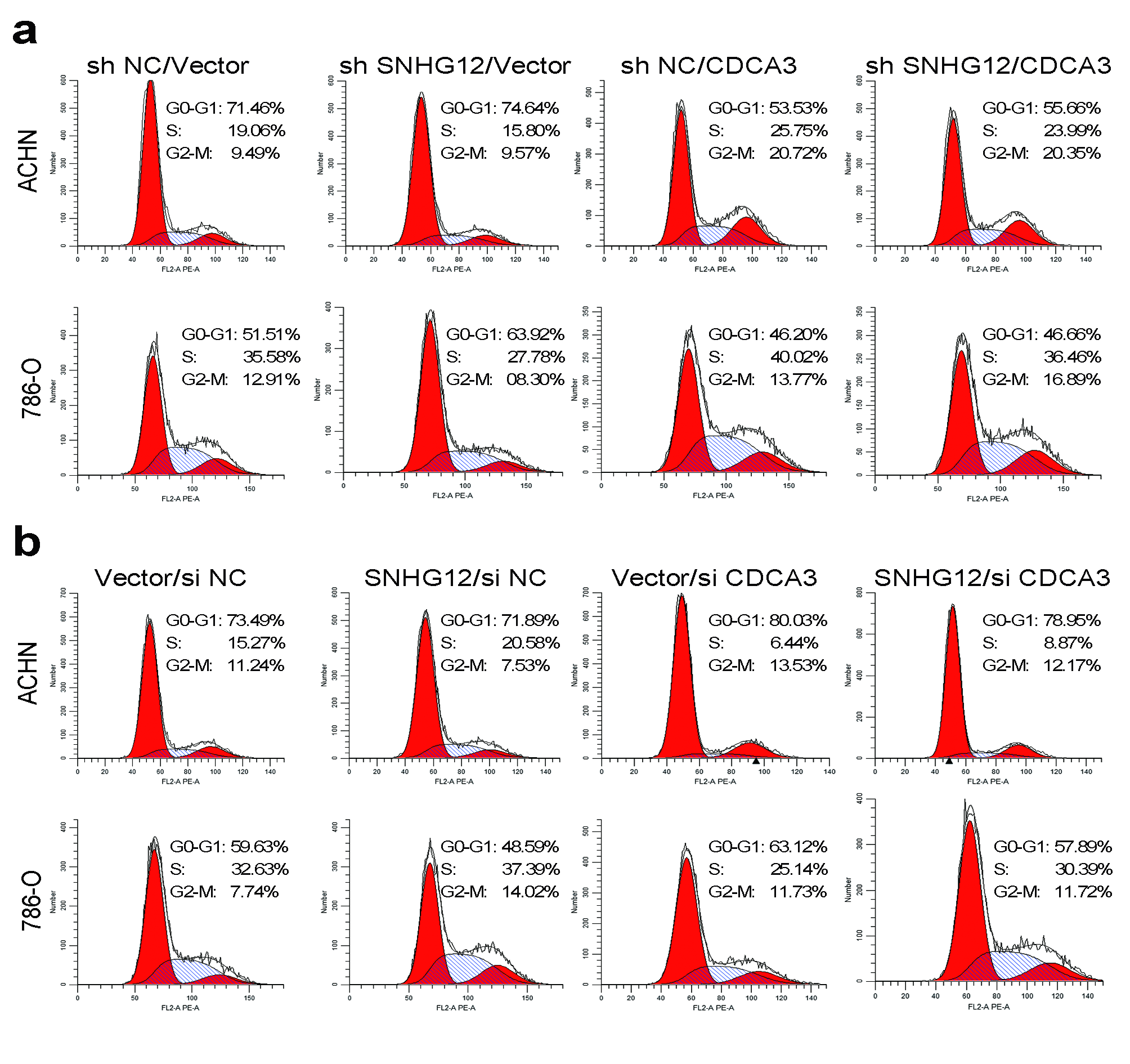

Supplement: Supplementary file 6 — Supplementary Figure 5 [file 41419_2020_2713_MOESM6_ESM.tif]

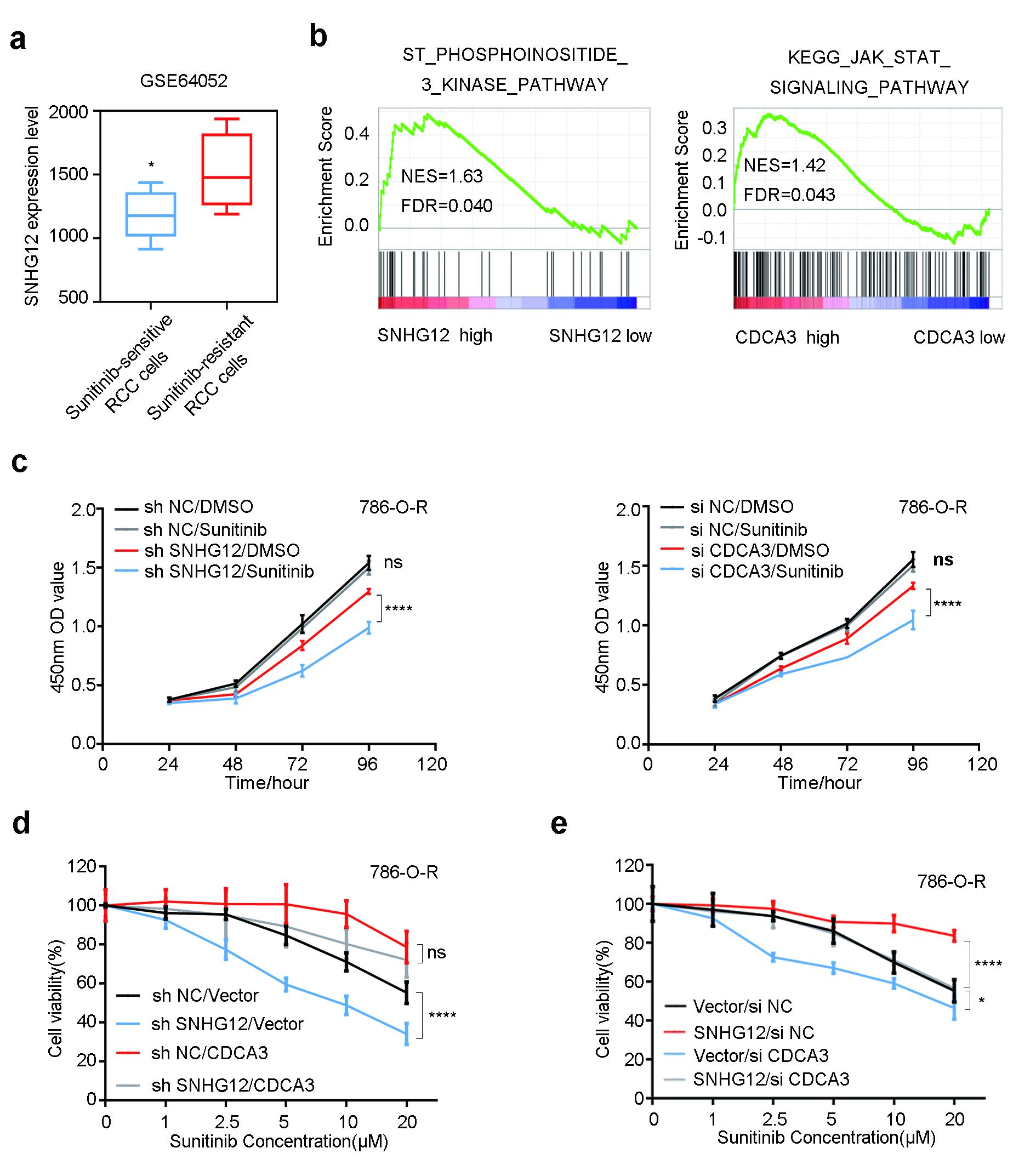

Supplement: Supplementary file 7 — Supplementary Figure 6 [file 41419_2020_2713_MOESM7_ESM.tif]

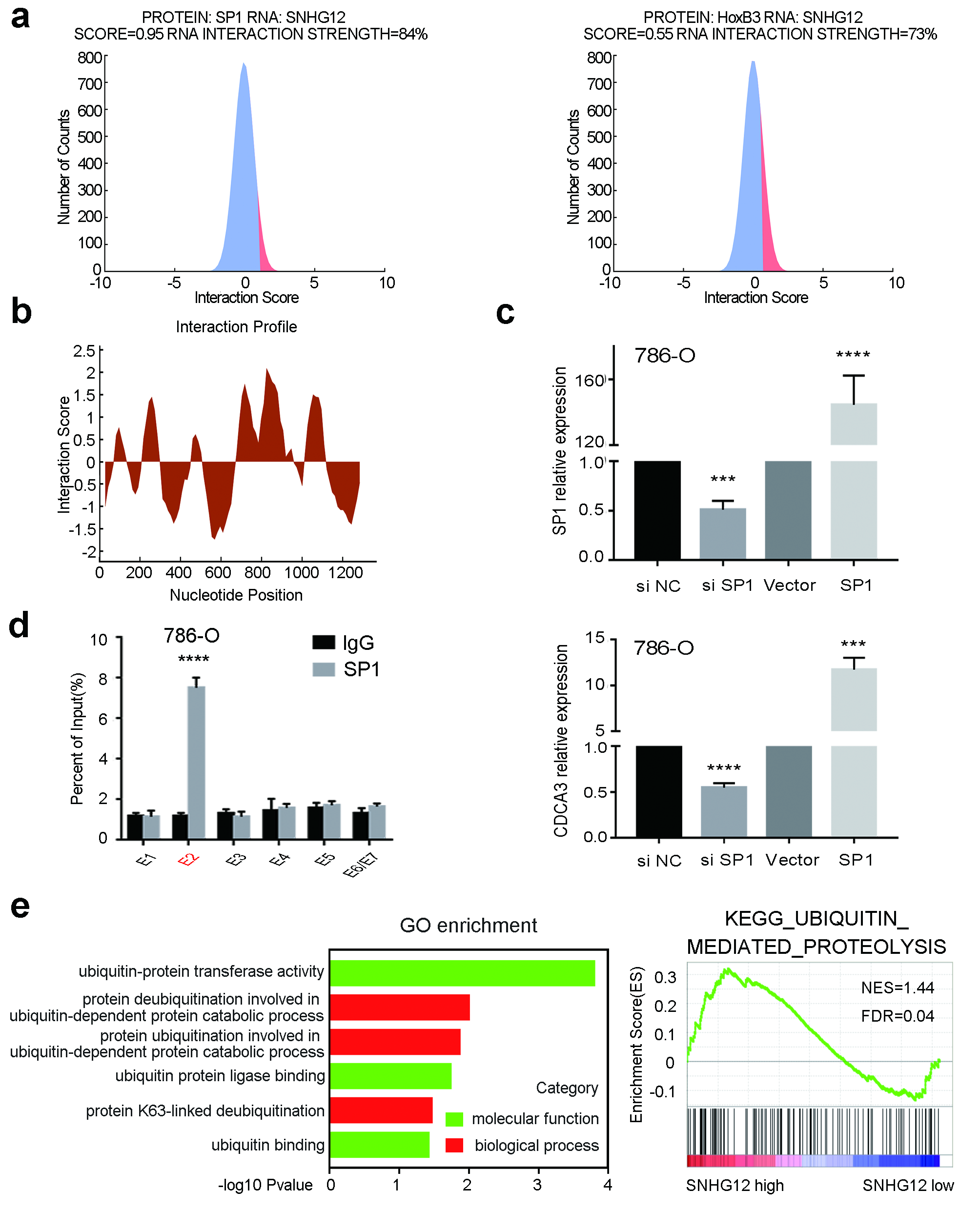

Supplement: Supplementary file 8 — Supplementary Figure 7 [file 41419_2020_2713_MOESM8_ESM.tif]
