# Supplementary material for: Long noncoding RNA SNHG12 promotes tumour progression and sunitinib resistance by upregulating CDCA3 in renal cell carcinoma
Source: Cell Death Dis. 2020 Jul 8;11(7):515. doi: 10.1038/s41419-020-2713-8 (PMC7343829; doi:10.1038/s41419-020-2713-8)
Supplement: Supplementary file 9 — Supplementary Figure legends [file 41419_2020_2713_MOESM9_ESM.docx]

**Supplementary Figure legends:**

**Supplementary Fig. 1** lncRNA MIR4435-2HG expression level was not related with clinicopathological factors, including G grade, T stage, lymph node metastasis and distant metastases.

**Supplementary Fig. 2** The distribution of SNHG12 was analyzed by FISH in ACHN cells. 18S and U6 showed cytoplasm and nucleus, respectively. Scale bars, 10 μm.

**Supplementary Fig. 3** The signaling pathways and genes correlated with SNHG12 according to GSAE analysis based on GEO database (GSE53757). Enrichment curves are shown for activated gene sets related to **a** EGFR signaling 24 h down, **b** cell cycle checkpoints, **c** Mesenchymal transition up, **d** DNA replication, **e** Metastasis down and **f** cell migration. FDR<0.25, *P*<0.05 was considered statistically significant. **g, h** The mRNA expression level of 6 candidate genes in 786-O cells with SNHG12 knockdown or overexpression. *P<0.05, **P<0.01, ***P<0.001. Error bars indicate mean ± SD.

**Supplementary Fig. 4** CDCA3 facilitated the proliferation, invasion and migration of RCC cells in vitro. **a** qRT-PCR and western blot assays were applied to analyze the expression level of CDCA3 after transfection by si CDCA3 or CDCA3 overexpression vector for 48 h in ACHN or 786-O cells. **b** Cell viability of ACHN and 786-O cells after knocking down or overexpressing CDCA3 was determined using CCK8 assays. **c, d** Transwell assays were performed in transfected ACHN and 786-O cells to evaluate cell migration and invasion ability (Magnification: 100X). **e, f** Cell cycle distribution was analyzed by PI staining in ACHN and 786-O cells after transfection by si RNA or overexpression plasmid for 48 h. *P<0.05, **P<0.01, ***P<0.001. Error bars indicate mean ± SD.

**Supplementary Fig. 5** SNHG12 regulated cell cycle via CDCA3 in RCC cells. **a, b** Cell cycle distribution was analyzed by PI staining in ACHN and 786-O cells after co-transfection by sh SNHG12 and CDCA3 overexpression vector or SNHG12 overexpression vector and si CDCA3 for 48 h.

**Supplementary Fig. 6** SNHG12 increased sunitinib resistance in RCC cells through CDCA3. **a** The expression level of SNHG12 in sunitinib-resistant RCC cells was higher than in sunitinib-sensitive RCC cells in GSE64052. **b** GSEA analysis for the correlation of PI3K/AKT signaling pathway and expression level of SNHG12 and CDCA3 according to GEO database (GSE53757. FDR<0. 25, P<0.05 was considered statistically significant). **c** In 786-O-R cells, knocking down SNHG12 or CDCA3 revered sunitinib resistance, as described by CCK-8 assays. **d,e** The sensitivities of 786-O-R after co-transfection by sh SNHG12 and CDCA3 overexpression vector or SNHG12 overexpression vector and si CDCA3 under different sunitinib concentration were determined by CCK-8 assay. *P<0.05, **P<0.01, ***P<0.001, ****P<0.0001. Error bars indicate mean ± SD.

**Supplementary Fig. 7** SNHG12 bound to and stabilized SP1 which activated CDCA3 transcription. **a** Interaction strength between SP1 and SNHG12 or HoxB3 and SNHG12 was predicted by *catRAPID* algorithm. **b** Potential SP1 binding sequences on SNHG12 was predicted by *catRAPID* algorithm. **c** qRT-PCR for mRNA levels of SP1 and CDCA3 in transfected 786-O cells. **d** ChIP-PCR assays were performed to show direct binding of SP1 to CDCA3 promoter regions in 786-O cells. **e** GO enrichment and GSEA analysis based on TCGA database. *P<0.05, **P<0.01, ***P<0.001, ****P<0.0001. Error bars indicate mean ± SD.
